# Aesthetic and Emotional Effects of Meter and Rhyme in Poetry

**DOI:** 10.3389/fpsyg.2013.00010

**Published:** 2013-01-31

**Authors:** Christian Obermeier, Winfried Menninghaus, Martin von Koppenfels, Tim Raettig, Maren Schmidt-Kassow, Sascha Otterbein, Sonja A. Kotz

**Affiliations:** ^1^Max Planck Institute for Human Cognitive and Brain Sciences, Department of NeuropsychologyLeipzig, Germany; ^2^Institute of Comparative Literature/Cluster of Languages of Emotion, Free University of BerlinBerlin, Germany; ^3^Institute of Comparative Literature, Ludwig Maximilians UniversityMunich, Germany; ^4^Institute of Medical Psychology, Goethe University FrankfurtFrankfurt, Germany

**Keywords:** meter, rhyme, emotion, aesthetics, cognitive fluency, poetry

## Abstract

Metrical patterning and rhyme are frequently employed in poetry but also in infant-directed speech, play, rites, and festive events. Drawing on four line-stanzas from nineteenth and twentieth German poetry that feature end rhyme and regular meter, the present study tested the hypothesis that meter and rhyme have an impact on aesthetic liking, emotional involvement, and affective valence attributions. Hypotheses that postulate such effects have been advocated ever since ancient rhetoric and poetics, yet they have barely been empirically tested. More recently, in the field of cognitive poetics, these traditional assumptions have been readopted into a general cognitive framework. In the present experiment, we tested the influence of meter and rhyme as well as their interaction with lexicality in the aesthetic and emotional perception of poetry. Participants listened to stanzas that were systematically modified with regard to meter and rhyme and rated them. Both rhyme and regular meter led to enhanced aesthetic appreciation, higher intensity in processing, and more positively perceived and felt emotions, with the latter finding being mediated by lexicality. Together these findings clearly show that both features significantly contribute to the aesthetic and emotional perception of poetry and thus confirm assumptions about their impact put forward by cognitive poetics. The present results are explained within the theoretical framework of cognitive fluency, which links structural features of poetry with aesthetic and emotional appraisal.

## Introduction

In infant-directed speech, play, religious, and social rites, festive events, and other social occasions, almost all cultures of the world use “special” language featuring the superimposition of metrical patterning and sound similarities of various types. The use of these “poetical” features of language can be traced back several 1000 years and is likely to predate by far the written record of human language. Although regular metrical patterns have been spurned in western poetry by many of the avant-garde authors of the twentieth century, recently regular meter as a stylistic feature has resurfaced even in cutting-edge poetry as a consequence of the “new orality” of movements such as “spoken word,” slam poetry, etc. Poetry proper is only one of the many uses of language “poeticized” or “rhetoricized” in this way. The current study draws on stanzas^1^ from lyrical poetry to investigate the aesthetic and emotional effects of meter and rhyme.

Two aspects of poetry may contribute to the emotional responses it may elicit: its lexical content and its structural features (i.e., poetic form). While there is substantial evidence that the valence of words can influence the way they are perceived and processed (e.g., Kuchinke et al., 2005; Kanske and Kotz, 2007), little is known about how certain formal features contribute to the aesthetic and emotional reception of poetry. Attempting to link poetic structure and its potential aesthetic or emotional effects is one of the central concerns in literary studies, but more recently also in cognitive research.

The idea that poetic structure influences the reception of poetry is not new. Since Greek antiquity, rhetoricians (most notably Gorgias as in Aristotle, 1926) and philosophers (e.g., Aristotle, 1932) debated how stylistic figures affect recipients. For instance, Aristotle in his work “*Poetics*” (1932, 1449b, 28–30) claimed that the “sweetness” of meter and sound harmony in the language of tragedy, specifically in its sung portions, is one of the reasons why we can take “pleasure” (gr. *hedoné*) even in tragic plots. Furthermore, he already emphasized that poetry reception can be linked to memory formation.

This rich tradition of rhetoric and poetics has influenced literary studies on rhetoric and poetry ever since. For example, Jakobson (1960) essentially referred to these well-established concepts and further specified them in his seminal work on the processing of poetical language. Yet like most of traditional rhetoric and poetics, Jakobson did not try to account for the effects of poetical language in terms of general psychological mechanisms, be they primarily cognitive or affective in nature. By contrast, some proponents of cognitive poetics (e.g., Van Peer, 1990; Tsur, 2008) have made significant suggestions and have taken steps in this direction. Our study continues and deepens this approach; specifically, we suggest that the cognitive fluency-hypothesis (e.g., Reber et al., 2004) may be of substantial explanatory power for the features under study.

As described by Jakobson (1960), meter and rhyme are two of the most important and most characteristic features of poetry. Not surprisingly, these two features have received much interest from literary and language researchers alike. Though being quite different in form, both features rely on a similar type of mechanism to structure poetry. They represent patterns of recurrence or similarity, i.e., both features structure poetry periodically in time (e.g., Turner and Pöppel, 1983; Lerdahl, 2001).

Rhymes represent pairs of words that are phonologically identical from the last accented vowel to the end of a word (e.g., ball/fall; Fabb, 1997, p.118). Besides their potential effect on aesthetic experience, they have been claimed to influence recall and comprehension of words (e.g., Lea et al., 2008). For instance, several studies have shown that target words rhyming with a preceding prime word are easier to process than non-rhyming target words (e.g., Rugg, 1984a,b; Kramer and Donchin, 1987; Coch et al., 2005). Furthermore, rhymes seem to contribute to the organization of lexico-semantic information in the mental lexicon (e.g., Allopenna et al., 1998). In a word recognition experiment, the authors reported that not only target words, but also the rhyming competitors were activated to a similar degree. This suggests that word cohorts with similar rhyme structure are activated in a comparable manner.

In poetry, (end) rhyme structures a poem at the level of the verse by strongly marking the ends of single verses and hence the onset of the caesura between two verses (cf. Turner and Pöppel, 1983). We suggest that rhyme – next to the already mentioned effects – highlights the overall metrical gestalt of a *verse*^2^, at least in poems of the type we used in the present experiment. Moreover, the phonological matching constitutive of rhyme produces a pattern of recurrence between two ante-caesura-syllables of different verses, which in turn makes the higher order gestalt of *stanzas* more predictable and memorable. In other words, end rhymes in metered poetry temporally structure both single verses and their configuration within the multi-verse unit of the stanza. They do so by placing additional emphasis on metrical patterning and by producing a phonological resonance between two selected words, or syllables, in the ante-caesura position, i.e., the final word/syllable of a verse (Fabb, 2009). In line with the widely held assumption that beauty relies on structures of similarity and recurrence, – such as symmetry and various patterns of repetition (Fechner, 1876; Berlyne, 1974; Garner, 1974) – rhyme can also be hypothesized to enhance the perceived “beauty” of a poem and hence its aesthetic liking. Finally, since antiquity rhetoric and poetics postulate that rhetorical elaboration of whatever sort should make the message of an utterance more salient and more emotionally involving.

Meter generally refers to the perception of alternating accented (strong) or unaccented (weak) syllables (Selkirk, 1986; Port, 2003). Regular meter can influence the saliency of a stimulus and draws a perceiver’s attention toward a specific stimulus (for details, see Large and Jones, 1999). For instance, the syllabotonic meter, which is the most common form of meter in English and German poetry, is defined both by the alternation of stressed and unstressed events within a metered foot and the number of stressed syllables in a verse line. Whereas rhyme structures poetry in a symmetrical way at a larger time scale, meter provides an asymmetric temporal marking of poetry on a smaller time scale (see Fabb, 2009). Its rhythmic recurrences (in the present case, in form of an iambic or a trochaic meter) help to structure a verse line in time (for more details on the role of poetic meter, see Turner and Pöppel, 1983).

There is substantial evidence that metrical patterning in many forms of poetry is beneficial to cognitive processes (e.g., see Cutler and Foss, 1977). Regular metrical structure, for instance, is easier to remember and to reproduce than irregular metrical structure (Essens and Povel, 1985). It plays a role in language acquisition (e.g., Jusczyk, 1999), syntactic (Schmidt-Kassow and Kotz, 2009), and semantic processing (e.g., Mattys and Samuel, 1997; Rothermich et al., 2012). A small number of studies (e.g., Magne et al., 2007; Schmidt-Kassow and Kotz, 2009; Luo and Zhou, 2010; Rothermich et al., 2010) also investigated the processing of metered and non-metered speech by means of event-related potentials (ERPs) of the electroencephalogram (EEG) because the high temporal resolution of the method allows monitoring effects of metrical regularity as a stimulus unfolds in time. Overall, these studies reported increased negative ERP response within 400 ms of a critical stimulus’ onset to non-metered compared to metered stimulus quality suggesting that metered stimuli may be associated with less cognitive processing demand than non-metered ones.

In summary, both rhyme and meter are associated with structuring perceptual input by drawing attention toward prosodic stimulus properties and facilitating cognitive processing. Ease of processing could result in a reduced working memory load as well as predictions of upcoming stimulus events. Furthermore, proponents of aesthetics and cognitive poetics postulate that similarity, symmetry, and other types of recursive patterning based on rhyme and metrical structure are basic features of beauty (e.g., Grammer and Thornhill, 1994; Rhodes et al., 1998; Jacobsen and Höfel, 2002; Jacobsen et al., 2006; Di Dio et al., 2007). Therefore, rhyme and the metrical structure of poetry should impact aesthetic liking and should also render poetry more emotionally involving. However, so far no systematic investigation has been undertaken to show how these two structural features of poetry as well as their interplay with lexical content impact the aesthetic and emotional processing of poetry^3^. We therefore set out to investigate whether there is a link between specific structural properties of poetry and aesthetic and emotional responses to it.

In order to address this question, we collected a set of 60 stanzas taken from nineteenth and twentieth century German poems. On the basis of these four verse stanzas we produced highly controlled versions that differed in lexicality (real words vs. pseudo-words), meter (metered vs. non-metered), and rhyme (rhyming vs. non-rhyming). Participants listened to the pre-recorded stanzas and rated them on four scales: liking (aesthetic appreciation), strength of emotional response (intensity), emotion perceived as represented, or expressed in the stanzas (perceived emotion), and emotion actually felt while listening to the stanzas (felt emotion). These four categories (aesthetic liking, intensity of being affected/involved, perceived emotion, felt emotion) were chosen based on the propositions provided by cognitive poetics and the cognitive fluency theory to differentiate between aesthetic (liking) and emotional effects (intensity, perceived, and felt emotion) based on rhyme and metrical structure. If the hypotheses put forward by classical rhetoric and cognitive poetics are correct, stylistic figures such as meter and rhyme should influence aesthetic and emotion ratings. More specifically, we should expect to find higher aesthetic value ratings, higher emotional intensity ratings, higher perceived emotion, and higher felt emotions ratings for rhyming as compared to non-rhyming stanzas. End rhymes are very salient temporal and phonological markers in poetry and therefore the presence/absence of a rhyme should lead to rather robust effects in all four rating categories. Similarly to the rhyme manipulation, we would also expect effects of meter on all four rating categories based on propositions put forward by the cognitive fluency theory. Specifically, we would expect higher rating for metered as compared to non-metered stanzas. However, the meter manipulation, while perceived and detected by participants in a pretest, could also be perceived as less salient compared to the rhyme manipulation. As a consequence, effects of meter in the different rating categories could be weaker. In order to show clear effects of stylistic features on listener’s aesthetic and emotional reception of poetry, expected effects should also show independent of lexicality. If structural features interact with lexico-semantic content of poems, this would falsify the proposition put forward by the cognitive fluency theory that structural features are important contributors to aesthetic liking and emotional responses *per se*. However, based on evidence from emotion research we expect lexicality to impact the emotion ratings, as it is known that the valence of a word can influence emotional responses to it (e.g., Kuchinke et al., 2005; Kanske and Kotz, 2007). The aesthetic liking rating, however, should be rather unaffected, as it should only capture the stylistic quality of the stanzas. As this study is of exploratory nature, it is difficult to clearly predict potential interactions of the factors. However, based on assumptions in cognitive poetics, both stylistic factors and semantic content should contribute to the emotional response in a perceiver. Therefore it is reasonable to assume that those ratings that are concerned with the perceived and felt emotional content of the stanzas may be specifically prone to interaction between lexicality and the stylistic factors.

## Materials and Methods

### Participants

Nineteen native German-speaking participants were paid to participate and signed a written informed consent following the guidelines of the Ethics committee of the University of Leipzig. Two participants were excluded from further statistical analysis due to technical problems during data collection. The remaining 17 (11 female; 19–30 years, mean 24.2 years) were right-handed (mean laterality coefficient 87.6, Oldfield, 1971), had normal or corrected-to-normal vision, no known hearing deficits, and had not taken part in the pre-testing of the stimulus material.

### Stimuli

The basic stimulus set contained 100 four-verse stanzas from nineteenth and early twentieth century German poetry (e.g., *Abendständchen* by Brentano, 1803). These samples usually constituted the first stanza of the respective poems. All stanzas belonged to an elementary and well-known type of stanza, the so-called German “Volksliedstrophe” (roughly equivalent to the English ballad stanza, for an example see Table 1). The stimuli were controlled for metrical form (iambic vs. trochaic), stanza scheme (isometric), rhyme scheme (half of the stanzas contained rhyming couplets, the other half contained alternating rhymes), syntactic regularity, length of the verses (85–125 letters per verse), the absence of enjambments as well as syntactic ellipses. Only nouns and verbs were accepted in the rhyming position. We also excluded poems that were too well-known to control for familiarity. For each of the 100 stanzas four different versions were constructed based on the factors METER (metered vs. non-metered) and RHYME (rhyming vs. non-rhyming). The first version constituted the original stanza (metered/rhyming). The second version was a metered, yet non-rhyming version of the stanza (metered/non-rhyming). The third version was non-metered, but contained the original rhymes (non-metered/rhyming), whereas the fourth version was both non-metered and non-rhyming (non-metered/non-rhyming). The altered versions were constructed according to the following principles: the original words and word order in the stanzas were kept identical whenever possible. The non-metered versions were obtained by adding one or two syllables to each verse, e.g., by changing particles or function words, modifying adjectives or substituting nouns with different ones of the same meaning (see Table 2). For the non-rhyming versions, the first word of each rhyme pair was substituted. Apart from modifying rhyme and meter, great care was taken that other stylistic features, such as metaphor and syntactical figures (e.g., anaphora) were kept identical (see Table 1).

**Table 1:** **Stimulus examples (Brentano, 1803: *Abendständchen*)**.

Real word stanzas	Pseudo-word stanzas
**Original version (metered/rhyming)**	**Metered/rhyming**

Holdes Bitten, mild Verlangen,	horbef dickel, lirg selmanem,
wie es süß zum Herzen spricht!	hie esch wüss psul helpsem strift!
Durch die Nacht, die mich umfangen,	gulf bie lask, bie lis unschalem,
blickt zu mir der Töne Licht!	drickt pfu nil bel pöle rift!

**Metered/non-rhyming**	**Metered/non-rhyming**

Holdes Bitten, mild Begehren,	horbef dickel, lirg gedehlel,
wie es süß dem Herzen klingt!	hie esch wüss bel helpsem trimp!
Durch die Nacht, die mich umfangen,	gulf bie lask, bie lis unschalem,
blickt zu mir der Töne Licht!	drickt pfu nil bel pöle rift!

**Non-metered/rhyming**	**Non-metered/rhyming**

Holdes Bitten, mildes Verlangen,	horbef dickel, linbech selmanem,
wie es süß mir zum Herzen spricht!	hie esch wüss nil psul helpsem strift!
Durch die Dunkelheit, die mich umfangen,	gulf bie gulpenheip, bie lis unschalem,
blickt zu mir dieser Töne Licht!	drickt pfu nil biechel pöle rift!

**Non-metered/non-rhyming**	**Non-metered/non-rhyming**

Holdes Bitten, mildes Begehren,	horbef dickel, linbech gedehlel,
wie es süß mir im Herzen klingt!	hie esch wüss nil psul helpsem trimp!
Durch die Dunkelheit, die mich umfangen,	gulf bie gulpenheip, bie lis unschalem,
blickt zu mir dieser Töne Licht!	drickt pfu nil biechel pöle rift!

**Table 2:** **Example for the modification of meter and rhyme in the stimulus material (Brentano, 1803: *Abendständchen*)**.

Original stanza(metered/rhyming)	Non-metered/non-rhyming version of the stanza	Manipulation
Holdes Bitten, *mild Verlangen*,	Holdes Bitten, *mildes Begehren*,	Modification of adjective, substitution of noun
wie es süß *zum Herzen spricht*!	wie es süß *mir im Herzen klingt*!	Addition of pronoun, substitution of preposition, and verb
Durch die *Nacht*, die mich umfangen,	Durch die *Dunkelheit*, die mich umfangen,	Substitution of noun
blickt zu mir der Töne Licht!	blickt zu mir dieser Töne Licht!	Modification of article

*The modified words are represented in italics*.

Additionally, for each of the four versions of a stanza, a pseudo-word version was created (factor LEXICALITY: real words vs. pseudo-words). The pseudo-words were constructed by substituting the original consonants with different ones while keeping vowels constant. German phonotactic rules were considered to guarantee the pronounceability of the pseudo-word verses (for more details on the pseudo-word construction, see Raettig and Kotz, 2008).

In summary, 8 versions were created for each of the 100 original stanzas based on the factors LEXICALITY, METER, and RHYME resulting in an experimental set of 800 stanzas. A professional actor produced the stanza versions with natural intonation. Each stanza was recorded several times and the best sounding recordings were subsequently chosen and normalized to 78 dB to minimize differences in intensity between the stanzas. Furthermore, separate acoustic analyses of duration, maximal pitch, minimal pitch, and mean pitch were calculated to ensure that critical parts of the different verse versions, i.e., the last word of each line (for which one would assume the maximal effect of the stylistic features), did not differ in terms of acoustic properties (see Table 3).

**Table 3:** **Statistical values (mean, range) across the critical, final words of each line for all stimulus conditions**.

			Mean pitch (Hz)	Max pitch (Hz)	Min pitch (Hz)	Duration (s)
Real words	Metered	Rhyming	84.30 (range: 79.07)	95.66 (range: 142.81)	74.36 (range: 65.33)	0.51 (range: 0.69)
		Non-rhyming	84.02 (range: 62.38)	94.31(range: 79.82)	74.54 (range: 83.69)	0.51 (range: 0.75)
	Non-metered	Rhyming	82.89 (range: 127.71)	97.29 (range: 175.27)	69.50 (range: 104.02)	0.51 (range: 0.71)
		Non-rhyming	82.90 (range: 108.51)	95.79 (range: 153.57)	69.52 (range: 104.97)	0.51 (range: 0.80)
Pseudo-words	Metered	Rhyming	83.63 (range: 57.95)	94.43 (range: 113.88)	73.85 (range: 80.52)	0.51 (range: 0.66)
		Non-rhyming	83.38 (range: 54.70)	93.52 (range: 79.48)	74.24 (range: 79.04)	0.51 (range: 0.72)
	Non-metered	Rhyming	81.46 (range: 86.72)	94.83 (range: 147.94)	69.10 (range: 87.45)	0.51 (range: 0.66)
		Non-rhyming	81.80 (range: 111.68)	94.30 (range: 131.80)	69.58 (range: 102.12)	0.52 (range: 0.77)

### Pretest

In order to verify and optimize the quality of the meter manipulation, a rating study was performed. Forty native speakers of German were asked to rate a subset of the stimuli (real word stanzas, which were either metered/rhyming or non-metered/rhyming) for their rhythmic regularity on a five-point Likert-scale (1 – very irregular to 5 – very regular). Metered stanzas were judged to be significantly more regular than non-metered stanzas [*F*(1, 39) = 189.7, MSE = 0.154, *p* < 0.0001]. Based on the results of the rating, 30 stanzas with rhyming couplets as well as 30 stanzas with alternating rhymes were selected for a final stimulus set, which showed the largest difference in the rhythmic regularity rating for the metered and non-metered versions. Thus, the resulting final stimulus set contained 480 stanzas (60 stanzas × 8 versions, for a list of all stanzas used in the present experiment, see Table A1 in Appendix).

### Procedure

Participants listened to each stanza version via headphones. They were instructed to rate the stanzas spontaneously along four dimensions: liking, intensity, perceived emotion, and felt emotion. For the liking rating, they were told to judge the overall aesthetic effect of the stanza taking into account the tonal and rhythmic properties (five-point Likert-scale: 1 – very bad to 5 – very good). For the intensity rating, participants had to assess the strength of the emotional response to the stanzas (five-point Likert-scale: 1 – very weak to 5-very strong). On the perceived emotion scale participants had to indicate the emotion they perceived as represented or expressed in the stanzas (five-point Likert-scale: 1 – very negative to 5 – very positive), whereas they had to rate the emotion they actually experienced while listening to the stanzas on the felt emotion scale (five-point Likert-scale: 1 – very negative to 5 – very positive).

The rating took place in two sessions. In the first session, participants judged the pseudo-word versions of the stanzas (240 poems, i.e., 60 stanzas in each of the four conditions: metrical/rhyming, metrical/non-rhyming, non-metrical/rhyming, non-metrical/non-rhyming), followed by a second session, in which they rated the real word stanzas (again 240 verse). In each session the stanzas were presented in mini-blocks of six stanzas of the same type, all in all resulting in 40 mini-blocks per session. The whole rating lasted approximately 2.5–3 h.

### Data analysis

Each of the different ratings was subjected to a repeated-measures ANOVA with the factors lexicality (real words vs. pseudo-words), meter (metrical vs. non-metrical), and rhyme (rhyming vs. non-rhyming).

## Results

**Figure 1:**
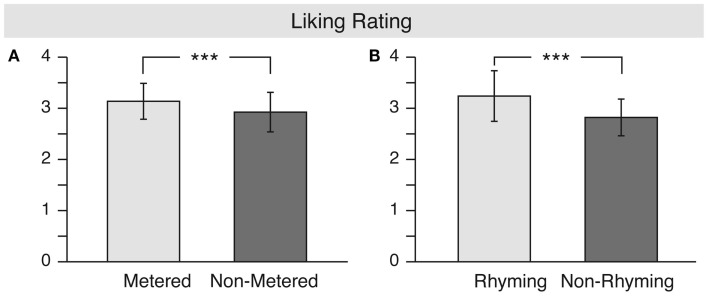
**Results for the “liking” rating**. The **(A)** shows the main effect of meter, whereas the **(B)** shows the main effect of rhyme.

For the liking ratings (see Figure 1), the ANOVA revealed a significant main effect of meter [*F*(1, 16) = 17.7, MSE = 0.084, *p* = 0.001, $\eta_{p}^{2}=0.525$] and a main effect of rhyme [*F*(1, 16) = 13.2, MSE = 0.439, *p* = 0.002, $\eta_{p}^{2}=0.452$], indicating that both metered as well as rhyming stanzas were reliably rated as more aesthetically pleasing than non-metered or non-rhyming stanzas.

**Figure 2:**
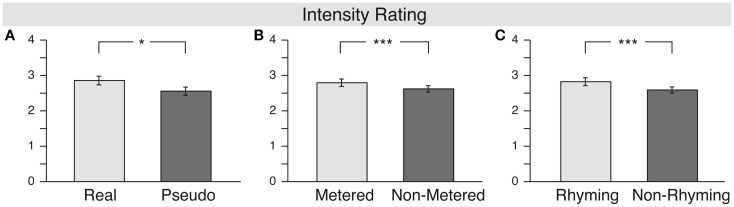
**Results for the “intensity” rating**. The **(A)** illustrates the main effect of lexicality, the middle one **(B)** the main effect of meter, and the right one **(C)** shows the main effect of rhyme.

The analysis of the intensity ratings (see Figure 2) also confirmed significant main effects of meter [*F*(1, 16) = 12.7, MSE = 0.081, *p* = 0.003, $\eta_{p}^{2}=0.443$] and rhyme [*F*(1, 16) = 15.2, MSE = 0.123, *p* = 0.001, $\eta_{p}^{2}=0.488$], but also a significant effect of lexicality [*F*(1, 16) = 4.5, MSE = 0.677, *p* = 0.05, $\eta_{p}^{2}=0.220$] and a marginally significant two-way interaction of meter and rhyme [*F*(1, 16) = 3.9, MSE = 0.008, *p* = 0.065, $\eta_{p}^{2}=0.198$]. This result suggests that not only meter and rhyme, but also the lexicality of a stanza influences the strength of the emotional response to a stanza. Specifically, the emotional response to real word stanzas was stronger than that to pseudo-word stanzas.

**Figure 3:**
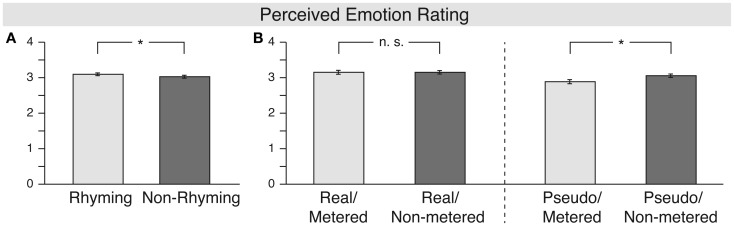
**Results for the “perceived emotion” rating**. The **(A)** shows the main effect of rhyme, whereas the **(B)** depicts the interaction of lexicality and meter, with the left part showing the main effect of meter for real world stanzas and the right part showing the main effect of meter for pseudo-word stanzas.

The analysis of perceived emotion (see Figure 3) revealed significant main effects for lexicality [*F*(1, 16) = 9.4, MSE = 0.117, *p* = 0.007, $\eta_{p}^{2}=0.371$], meter [*F*(1, 16) = 6.6, MSE = 0.036, *p* = 0.02, $\eta_{p}^{2}=0.293$], and rhyme [*F*(1, 16) = 7.9, MSE = 0.020, *p* = 0.012, $\eta_{p}^{2}=0.332$]. The main effects of lexicality and meter were specified by a significant two-way interaction of both factors [*F*(1, 16) = 8.3, MSE = 0.029, *p* = 0.011, $\eta_{p}^{2}=0.344$]. Resolving the interaction showed that non-metered pseudo-word stanzas were rated significantly more positive than metered pseudo-word stanzas [paired-*t*(16) = 2.83, *p* = 0.012, Cohen’s *d* = −0.770], whereas there was no such difference for the real word stanzas [paired-*t*(16) = 0.11; *p* = 0.99, Cohen’s *d* = 0.0009].

In summary, rhyming stanzas seem to be perceived more positively than non-rhyming ones. In contrast, on the scale of perceived emotion, meter only had an effect in the pseudo-word stanzas. Interestingly, non-metered stanzas elicited more positive ratings than metered ones on this scale.

**Figure 4:**
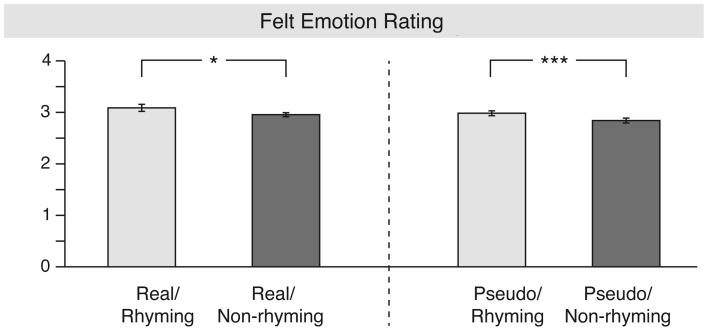
**Results for the “felt emotion” rating**. The left part shows the main effect of rhyme for the real word stanzas, whereas the right part depicts the main effect of meter for the pseudo-word stanzas.

On the scale of felt emotion (see Figure 4), statistical analysis revealed a significant main effect of rhyme [*F*(1, 16) = 12.8, MSE = 0.050, *p* = 0.002, $\eta_{p}^{2}=0.446$], and a significant three-way interaction of lexicality, meter, and rhyme [*F*(1, 16) = 4.8, MSE = 0.008, *p* = 0.05, $\eta_{p}^{2}=0.219$]. A step-down analysis of this interaction showed a significant main effect of rhyme. Rhyming stanzas were judged as eliciting a more positive feeling (“felt emotion”) in the perceiver than non-rhyming ones; this effect was stronger in the pseudo-word [*F*(1, 16) = 15.1, MSE = 0.011, *p* = 0.001, $\eta_{p}^{2}=0.487$, Cohen’s *f*^2^ = 0.73] than in the real word stanzas [*F*(1, 16) = 5.1, MSE = 0.030, *p* = 0.04, $\eta_{p}^{2}=0.241$, Cohen’s *f*^2^ = 0.59]. Felt emotion, therefore, only seemed to be influenced by rhyme in a sense that rhyming stanzas elicited a more positive emotional response than non-rhyming ones regardless of the varying contents of the stanzas. This effect was stronger in the pseudo-word than real word stanzas.

## General Discussion

The current study set out to investigate whether lexicality, meter, and rhyme as core components of poetry influence the aesthetic and emotional response to poetry in non-expert listeners. For this purpose, participants listened to a set of eight different versions of stanzas from nineteenth and early twentieth century German poetry and judged them on four different scales: liking, intensity, perceived emotion, and felt emotion. Rhyme significantly influenced liking, intensity, perceived emotion as well as felt emotion ratings, with rhyming stanzas leading to a more positive aesthetic and emotional evaluation. Similarly, regular meter enhanced aesthetic liking and more intense emotional processing as compared to the non-metered versions of the stanzas. In contrast to meter and rhyme, lexicality, as hypothesized, did not impact the overall aesthetic appraisal of the stanzas, but only influenced the emotional ratings, i.e., the emotional intensity and perceived emotion ratings with real word stanzas eliciting more positive responses than pseudo-word stanzas. All main effects are in line with our proposed hypotheses and provide first empirical evidence in support of the assumption that the aesthetic evaluation of poetry relies mainly on the stylistic features of the respective stanzas independent of semantics. Additionally, these findings lend support to the assumptions put forth by cognitive fluency theory, i.e., that structural features impact aesthetic and emotional processing.

Besides the reported main effects two interactions warrant a closer look and discussion. First, lexicality meter, and rhyme interact and influence perceived emotion ratings. This is in line with one of the assumption of cognitive poetics that both stylistic factors and semantic content should contribute to the emotional response in a perceiver. However, when resolving this interaction, only a quantitative difference for rhyme remained at different levels of lexicality. Specifically, rhyming stanzas elicited more positive ratings than non-rhyming ones. This effect was stronger for pseudo-word stanzas than for real word stanzas suggesting that lexicality indeed affects the emotional response to poetry. Possibly, rhyme exhibits a stronger influence on felt emotion if there is no meaning that can interfere with its effect. More importantly, however, the effect of rhyme in the felt emotion rating may also reflect the higher saliency of rhyme in the stimulus material as compared to the meter manipulation, as we did not find any effects of meter in the felt emotion ratings. Again, this result is in line with our hypotheses. A potential explanation for the absence of a similar effect for meter may well be due to the fact that we used only single stanzas. In all likelihood, the effect of meter grows stronger over time, and units of four verses may simply be too short for meter to take hold of the reader. Second, we also found a significant interaction between lexicality and meter for the perceived emotion ratings (i.e., emotion detected as represented or expressed in the stanzas). Whereas there was no effect of meter in the real word stanzas – most likely because lexicality strongly contributes to the emotion we attribute to a poem, – non-metered pseudo-word stanzas surprisingly elicited more positively perceived emotions than metered pseudo-word stanzas. One may conclude that this particular effect results from a perceived parallel between form and content (the absence of metrical structure somehow “matching” the chaotic impression of the pseudo-words), but this is purely conjectural.

Taken together, the present study, to the authors’ knowledge, provides the first systematic experimental evidence that structural features of poetry, such as rhyme and meter, influence the aesthetic and emotional evaluation of poetic stanzas in naive (or non-expert) listeners. Thereby, the findings provide first experimental evidence for key assumptions put forward both by classical rhetoric and more recent cognitive poetics: the processing of a poem by a listener is indeed linked to its poetic structure. Interestingly, rhyme and meter that structure poetry at different temporal levels seem to affect the ratings rather independently, suggesting that they may also affect different cognitive processes. The research leaves open the question to what extent the present findings extend beyond lyrical poetry to other uses of meter and rhyme in speech and language. Potentially, the present findings could also be of broad relevance for spoken language perception *per se*. Temporal patterning based on metrical structure is present in spoken language and primarily referred to as prosody. Though the metrical structuring may not be as obvious as in poetic stanzas, there is little doubt that it is used in rhetorical persuasion or advertisement and has significant impact on the cognitive and emotional processing of communicative messages. Furthermore, the use of temporal structuring also goes far beyond the spoken word as we use communicative mimics, postures, and gestures. Therefore, the present data provides first evidence to further investigate how temporally coded paralinguistic factors contribute and interact in interpersonal communication and the cognitive and emotional processes underlying it.

Although the present data are in line with the propositions made by cognitive poetics, they cannot clarify two important and open issues that have not been satisfactorily addressed by cognitive poetics so far: what kind of cognitive processes are involved when listening to poetry and how are certain structures in poetry linked to either a positive or negative evaluation of a poem by the perceiver?

At the latest since the nineteenth century, hedonic valence has been psychologically interpreted along the lines of “cognitive expenditure” or the “principle of minimizing processing expenses” (Fechner, 1876), or, positively, of processing “ease” (“Bequemlichkeit;” for a brief survey on pertinent hypotheses in nineteenth century music psychology, see Stumpf, 1885). Today’s cognitive fluency theory (for a comprehensive review see Reber et al., 2004) adopts these ideas and combines them with Gestalt psychology (e.g., Koffka, 1935; Arnheim, 1974; Gombrich, 1984). According to this framework, the aesthetic experience of poetry depends on the perceiver’s processing dynamics, more specifically “the more fluent the perceiver can process an object, the more positive is his or her aesthetic response” (Reber et al., 2004, p. 365). Importantly, it is not the experience of fluency itself that leads to the liking of dot patterns, faces, paintings, etc., but the affective response due to the facilitated processing of a stimulus (Winkielmann and Fazendeiro, 2003, unpublished manuscript). In other words, cognitive fluency serves as a basis for the liking of a stimulus or an object. Therefore, it is important to identify which factors influence the degree of cognitive fluency. Previous research has shown that it depends both on the idiosyncratic processing experience of the recipient as well as on the features of the object to be processed. Using visual stimulation (e.g., patterns, faces, letters, etc.) researchers have identified various features that increase fluency such as informational content and complexity (e.g., symmetric shapes are easier to process than asymmetric shapes, Garner, 1974; see also Berlyne, 1974), contrast and clarity (Gombrich, 1984, 1995; Solso, 1997), balance and proportion (Fechner, 1876; Arnheim, 1974; Gombrich, 1995), and symmetry (e.g., Palmer and Hemenway, 1978; Royer, 1981; Palmer, 1991). For instance, Palmer and Hemenway (1978) presented letters that were either vertically or horizontally mirrored. Measuring reaction times, the authors were able to show that vertical symmetry is easier (faster) to detect than horizontal symmetry. I.e., vertically symmetric letters were processed with higher cognitive fluency than horizontally symmetric letters, which in turn led to a more positive experience (for details, see Winkielman et al., 2003) and aesthetic appreciation for the vertically symmetric letters^4^.

Note that cognitive fluency research so far has almost exclusively focused on visual stimuli. There is only a small amount of auditory perception studies that have been interpreted within the cognitive fluency framework (e.g., Schellenberg and Trehub, 1996). Nevertheless, as stated above, features such as meter and rhyme also represent patterns of recurrence similar to dot patterns or letters, which in general should also affect the ease of cognitive processing based on familiarity, clarity, or symmetry. In fact, some studies have already shown facilitatory effects on cognitive processing for both rhyme and meter (meter: e.g., Magne et al., 2007; Schmidt-Kassow and Kotz, 2009; Rothermich et al., 2010, 2012; rhyme: e.g., Rugg, 1984a,b; Kramer and Donchin, 1987; Coch et al., 2005). For instance, previous ERP studies have shown that rhyming word pairs are easier to process than non-rhyming word pairs (e.g., Rugg, 1984a,b) as shown by a smaller N400 response that is linked to the integration of semantic information into a context. Similarly, regular meter eases word list or sentence processing in comparison to irregular meter (e.g., Magne et al., 2007; Schmidt-Kassow and Kotz, 2009; Rothermich et al., 2010, 2012). Based on such findings, it is likely that rhyme and meter influence the evaluation of poetry by mechanisms put forward in the cognitive fluency theory. More precisely, rhyme and (regular) meter ease the cognitive processing of a poem and consequently the respective poem receives more positive aesthetic and emotional appraisal.

However, to really understand the cognitive processes involved in poetry perception and their relation to the aesthetic and emotional consequences elicited in the perceiver, one has to investigate its neural basis by means of imaging techniques (EEG, MEG, fMRI). Such a neuroaesthetic approach has already been successfully applied to the processing of visual art and music (for a review, see Chatterjee, 2011). Therefore, we call for the neuroaesthetics of poetry to elucidate the cognitive processes involved in poetic reception^5^ and their relation to the aesthetic and emotional response in the perceiver.

## Conclusion

The present rating study provides first experimental evidence that stylistic and structural devices such as meter and rhyme influence aesthetic and emotional responses to poetry. Specifically, regular meter and rhyme lead to a heightened aesthetic appreciation and intensity of processing as well as more positive emotional responses. A potential cognitive account for the present findings is provided by the cognitive fluency theory. However, neuroscientific investigations are needed to provide specific insight into the underlying neural and cognitive basis of such findings. We therefore propose a neuroaesthetic approach to investigate poetry reception.

## Conflict of Interest Statement

The authors declare that the research was conducted in the absence of any commercial or financial relationships that could be construed as a potential conflict of interest.

## Footnotes

^1^A stanza is one of various groups of verses that form a poem. The stanza is defined by poetic features such as a fixed number of verses and a fixed metrical pattern and/or rhyme scheme. It is usually marked off by a space.

^2^A verse is the basic metrical unit of a poem, acoustically marked by a caesura, graphically marked by a line break (for a cognitively oriented definition of the verse or single “line” of poetry see Turner and Pöppel, 1983).

^3^Note that there are a few studies, in which experts rated single poems (e.g., Tsur, 2008). It is known that production and reception of poetry is influenced by expertise (Peskin, 1998). In this paper, we are not investigating expertise or inter-individual differences in poetry reception, though these are certainly important issues. Rather we want to clarify whether the specific structure of poetry generally affects the processing of poems in student listeners, who are not experts based on their field of study, but have a comparable, general sense that meter and rhythm are stylistic features in poetry as part of basic school education.

^4^Note that these association and evaluation processes can be influenced by a perceiver’s personal prior experience.

^5^In a second step, this approach could also be extended to rhetoric speech and advertisement.

## Appendix

**Table A1:** **Stimulus material – list of stanzas**.

Author	Stanza
Avenarius	Abend
Brentano	Abendständchen
Brentano	Am Tage vor dem Abendmahl
Brentano	Ich wollt‘ ein Sträußlein binden
Bürger	Resignation. Nach der Rowe
Bürger	Robert
Celan	Ich trau mich nicht mehr mit Flöten
Dauthendey	Immer neue Küsse gib
Eggert-Schwarten	Ausschnitt aus Gegenwart
Eichendorff	Der frohe Wandersmann
Eichendorff	Der Morgen
Eichendorff	Klage
Fontane	Trost
Fontane	Wer schaffen will, muß fröhlich sein
George	Es zuckt aus grauem wolkenzelt
George	Geführt vom sang der leis sich schlang
Gleim	Daphne an den Westwind
Goethe	An Geheimerat von Willemer
Goethe	An Silvien
Goethe	Leichte Silberwolken schweben
Hauff	Steh‘ ich in finstrer Mitternacht
Herwegh	An einen Bekannten, der einen Orden erhalten hatte
Hesse	Nacht im Odenwald
Hofmannsthal	Die Beiden
Keller	Nacht im Zeughaus. 1
Kleist	Hier von der Welt geschieden
Kolmar	Die Fremde
Kraus	Vor einem Springbrunnen
Lappe	Verheimlichung
Lenau	Blick in den Strom
Lenau	Mein Herz
Lenau	Veränderte Welt
Liliencron	Für und für
Liliencron	Ist das alles?
Loerke	Abend auf der Grand‘ Place in Brüssel
Marx	Darum laßt uns alles wagen
Meyer	Die Lautenstimmer
Miller	Der Frühling
Morgenstern	Nach Norden
Mörike	Begegnung
Rilke	Der Schauspieler
Rilke	Die Sprache der Blumen Stachelbeere (Ribes grossularia)
Rilke	Friedhofsgedanken
Rilke	Ich lieb ein pulsierendes Leben
Rilke	Vorwärts
Ringelnatz	Es war einmal ein Kannibale
Ringelnatz	Morgenwonne
Schiller	Der Antritt des neuen Jahrhunderts
Schiller	Für Sophie Nösselt
Schwab	Das Neckarthal bei Canstatt. Auf eine Landschaft von Steinkopf
Storm	Herbst
Storm	Herbst (2)
Storm	Im Herbste 1850
Storm	Liebesklage eines Verschmähten (2)
Tieck	An einen Liebenden im Frühling 1814
Tieck	Frühe Sorge
Uhland	An einem heitern Morgen
Wedekind	Frühling – Ilse
Werfel	Der Weltfreund versteht nicht zu altern
